# High rate of antibiotic resistance among pneumococci carried by healthy children in the eastern part of the Democratic Republic of the Congo

**DOI:** 10.1186/s12887-018-1332-3

**Published:** 2018-11-19

**Authors:** Archippe M. Birindwa, Matilda Emgård, Rickard Nordén, Ebba Samuelsson, Shadi Geravandi, Lucia Gonzales-Siles, Balthazar Muhigirwa, Théophile Kashosi, Eric Munguakonkwa, Jeanière T. Manegabe, Didace Cibicabene, Lambert Morisho, Benjamin Mwambanyi, Jacques Mirindi, Nadine Kabeza, Magnus Lindh, Rune Andersson, Susann Skovbjerg

**Affiliations:** 1 Department of Infectious Diseases, Institute of Biomedicine, University of Gothenburg, Gothenburg, Sweden; 2 Panzi Hospital, Bukavu, Democratic Republic of the Congo; 3 Université Evangélique en Afrique, Bukavu, Democratic Republic of the Congo; 4 CARe – Center for Antibiotic Resistance Research, Gothenburg University, Gothenburg, Sweden; 5 Hôpital Général de Référence de Panzi, BP: 266 Bukavu, DR Congo

**Keywords:** *Streptococcus pneumoniae*, Antibiotic resistance, DR Congo, Nasopharyngeal carriage, Children, PCV13

## Abstract

**Background:**

Pneumococcal conjugate vaccines have been introduced in the infant immunisation programmes in many countries to reduce the rate of fatal pneumococcal infections. In the Democratic Republic of the Congo (DR Congo) a 13-valent vaccine (PCV13) was introduced in 2013. Data on the burden of circulating pneumococci among children after this introduction are lacking. In this study, we aimed to determine the risk factors related to pneumococcal carriage in healthy Congolese children after the vaccine introduction and to assess the antibiotic resistance rates and serotype distribution among the isolated pneumococci.

**Methods:**

In 2014 and 2015, 794 healthy children aged one to 60 months attending health centres in the eastern part of DR Congo for immunisation or growth monitoring were included in the study. Data on socio-demographic and medical factors were collected by interviews with the children’s caregivers. Nasopharyngeal swabs were obtained from all the children for bacterial culture, and isolated pneumococci were further tested for antimicrobial resistance using disc diffusion tests and, when indicated, minimal inhibitory concentration (MIC) determination, and for serotype/serogroup by molecular testing.

**Results:**

The pneumococcal detection rate was 21%, being higher among children who had not received PCV13 vaccination, lived in rural areas, had an enclosed kitchen, were malnourished or presented with fever (*p* value < 0.05). The predominant serotypes were 19F, 11, 6A/B/C/D and 10A. More than 50% of the pneumococcal isolates belonged to a serotype/serogroup not included in PCV13.

Eighty per cent of the isolates were not susceptible to benzylpenicillin and non-susceptibility to ampicillin and ceftriaxone was also high (42 and 37% respectively). Almost all the isolates (94%) were resistant to trimethoprim-sulphamethoxazole, while 43% of the strains were resistant to ≥3 antibiotics.

**Conclusions:**

Our study shows alarmingly high levels of reduced susceptibility to commonly used antibiotics in pneumococci carried by healthy Congolese children. This highlights the importance of local antibiotic resistance surveillance and indicates the needs for the more appropriate use of antibiotics in the area. The results further indicate that improved living conditions are needed to reduce the pneumococcal burden, in addition to PCV13 vaccination.

**Electronic supplementary material:**

The online version of this article (10.1186/s12887-018-1332-3) contains supplementary material, which is available to authorized users.

## Background

*Streptococcus pneumoniae*, or the pneumococcus, is a leading bacterial cause of death in young children worldwide, mainly due to pneumonia [1, 2]. The bacterium is also an important pathogen in other community-acquired respiratory infections, including acute *otitis media*, and in invasive infections, such as meningitis and sepsis.

Pneumococcal infections are estimated to cause 11% of all deaths in children less than 5 years of age worldwide, with a disproportionate number of these deaths in low- and middle income countries [3]. In sub-Saharan Africa, where most of the under-five deaths occur, the leading cause of death is pneumonia and children under the age of 2 years are the most affected [3, 4].

The Democratic Republic of the Congo (DR Congo) is one of the countries with the highest mortality due to childhood pneumonia; in 2015, pneumonia was the leading cause of death under 5 years of age, killing 46,000 Congolese children [5, 6].

Many risk factors, including malnutrition, lack of pneumococcal immunisation, parental smoking and crowded living conditions, [7, 8], as well as exposure to smoke due to the household use of solid fuels has been associated with an increased risk of pneumonia in children [9, 10]. Women and children living in severe poverty have the greatest exposure to household air pollution [11]. In DR Congo, open fires are commonly used in rural villages, while charcoal stoves and electricity are more often used in the cities.

To lower the burden of severe pneumococcal infections among children, pneumococcal conjugate vaccines, covering up to 13 of 98 known pneumococcal serotypes [12], have been introduced in the infant vaccination programmes in many countries. The 13-valent conjugate pneumococcal vaccine (PCV13), containing the serotypes 1, 3, 4, 5, 6A, 6B, 7F, 9 V, 14, 18C, 19A, 19F and 23F, was introduced in DR Congo in 2013. A recent study from Kenya showed that the prevalence of vaccine serotypes was reduced by two-thirds in children younger than 5 years of age after the introduction of PCV10, suggesting that the conjugate vaccines will have substantial effects in reducing invasive pneumococcal disease in Africa [13]. However, in many countries, pneumococcal disease caused by non-vaccine serotypes has increased after the start of vaccination [14] and, in the Gambia, an increase in non-typeable serotypes was noted after the introduction of PCV13; the clinical significance of this finding is not known [15].

There are some reports on the carriage rate and serotype distribution of pneumococci in healthy sub-Saharan children before and after PCV13 vaccination [13, 16, 17], but there are no available data on the child population in DR Congo, either before or after the introduction of PCV13.

According to recommendations revised by the World Health Organisation (WHO) in 2014, oral amoxicillin is the drug of choice for children with pneumonia, while parenteral ampicillin (or penicillin) together with gentamicin should be used in severe cases. Ceftriaxone is recommended as the second-line treatment in children with severe pneumonia who have failed with the first-line treatment. There might, however, be a delay of several years before these recommendations are implemented in local treatment guidelines. Since December 2016, the national policy in DR Congo recommends amoxicillin rather than trimethoprim-sulphamethoxazole (TMP-SMX) for the treatment of pneumonia at community level. As in many other low-income countries, the prescription of antibiotics is, however, not restricted solely to physicians, and children may be treated by people other than educated health workers [18]. Children hospitalised in the South-Kivu province, in eastern DR Congo, due to pneumonia are currently treated with ceftriaxone or ampicillin, together with gentamicin, according to local guidelines, while a combination of ceftriaxone and cloxacillin is used after 48 h without clinical improvement [19].

Resistance to antibiotics is a worldwide concern and the proportion of pneumococci that are not susceptible to penicillin even exceeds 50% in some countries [20]. Before the introduction of the conjugate vaccine, more than two thirds of the pneumococci detected in healthy children in Dar Es Salaam, Tanzania, were non-susceptible to penicillin [21], while the rate was 45% in Gambia [22]. A Peruvian study showed no changes in antibiotic resistance in colonising pneumococci after the introduction of the vaccine, suggesting significant antibiotic resistance in non–PCV7 strains [23], while other studies from South Africa [24], the United States of America [25] and Canada [26] have shown a decrease in the antibiotic resistance of invasive pneumococci.

Here, we report on the first study of nasopharyngeal carriage and predisposing conditions for pneumococcal colonisation in healthy Congolese children after the introduction of PCV13. The profiles of the circulating pneumococcal serotypes/serogroups and the antibiotic susceptibility of the carried pneumococci were also assessed.

## Methods

### Study population

From January 2014 to June 2015, 794 healthy children aged one to 60 months attending one of seven health centres in the South-Kivu province in the eastern part of DR Congo for immunisation or growth monitoring were included in the study. The health centres were located in the city of Bukavu (*n* = 3), in the suburban area (*n* = 1), or in the surrounding rural area (n = 3) (Additional file 1).

Written questionnaires about immunisation status and demographic factors were completed by trained final-year medical students or nurses in the presence of a paediatrician and a basic physical examination of the children was performed to monitor current signs of a respiratory tract infection. When available, the immunisation card was checked to confirm the vaccination status of the child. For the 284 healthy children enrolled in 2015, another questionnaire containing questions about socio-economic conditions and previous illness was added. The weight and height were measured and standardised for age using the Emergency Nutrition Assessment (ENA) software 2011 [27].

Signed informed consent was obtained from the parent or guardian of each included child. The study was approved by the Ethics Committees at the Université Catholique de Bukavu, DR Congo, and at the University of Gothenburg, Sweden.

### Specimen collection

A nasopharyngeal specimen was obtained from all participating children using an Eswab (Copan Diagnostics Inc., Murrieta, CA). A single trained investigator at each centre obtained the sample following a standardised procedure. The head of the child was tipped backwards and gently immobilised. The bent swab was inserted into the nostril and then passed into the nasopharynx to a distance equal to that from the nose to the tip of the ear and kept in that position for 5 s. The samples were shipped to the Clinical Laboratory at Panzi Hospital within two to 6 h for subsequent pneumococcal culture.

### Culture and antibiotic susceptibility testing of pneumococci

The samples were cultured for *Streptococcus pneumoniae* on 5% human blood agar plates (Oxoid Columbia Blood Agar Base – Thermo Fisher Scientific, Waltham, MA), incubated overnight at 34–36 °C in closed jars (Oxoid Limited, Thermo Fisher Scientific, Hampshire, UK) supplied with CO_2_ paper sachets (BD GasPak™ EZ CO_2_ Container System, Becton, Dickinson and Company, Franklin Lakes, New Jersey) and CO_2_ indicators (BD CO_2_ Indicator 0.5 mL, Becton, Dickinson and Company).

Suspected pneumococci were identified by a positive optochin test (diameter ≥ 14 mm) and were further tested for antibiotic susceptibility using a disc diffusion test against oxacillin (1 μg) (screening for beta-lactam resistance), trimethoprim-sulphamethoxazole (TMP-SMX) (1.25/23.75 μg), norfloxacin (10 μg) (screening for fluoroquinolone resistance, i.e. levofloxacin and moxifloxacin), tetracycline (30 μg), erythromycin (15 μg) and clindamycin (2 μg) (all from Oxoid Limited), using breakpoints according to the European Committee on Antimicrobial Susceptibility Testing, 2017 [28]. The bacteria and antibiotic discs were applied to Muller Hinton agar plates (Oxoid Limited) supplied with 5% sheep blood (Thermo Fisher Scientific) and 20 mg/L β-Nicotinamide adenine dinucleotide (NAD) (AppliChem GmbH, Darmstadt, Germany) that were incubated over night at 34–36 °C in a CO_2_ environment as described above. Pneumococcal isolates with reduced sensitivity to oxacillin (diameter < 20 mm) were further tested using minimal inhibitory concentration (MIC) determination against penicillin G, ampicillin and ceftriaxone (all 0.016–256 μg/mL, bioMérieux, Marcy l’Etoile, France). Pneumococci with an MIC of > 0.06 mg/L were defined as having reduced susceptibility to benzylpenicillin. Multi-drug resistant (MDR) isolates were defined as those that were non-susceptible (intermediate or resistant) to at least one drug from three or more different classes of antimicrobial agents, including the beta-lactams (the penicillins benzylpenicillin and ampicillin and the cephalosporin ceftriaxone) [29]. Apart from the beta-lactams, all drugs tested belong to different classes, namely fluoroquinolones (norfloxacin), lincosamides (clindamycin), macrolides (erythromycin), folate pathway inhibitors (trimethoprim-sulphamethoxazole) and tetracyclines (tetracycline) [29]. The isolates were frozen (− 20 °C) in STGG storage medium [30] before being transported to Gothenburg, Sweden, for further analyses.

### Reproducibility of the antibiotic susceptibility results

**Fig. 1:**
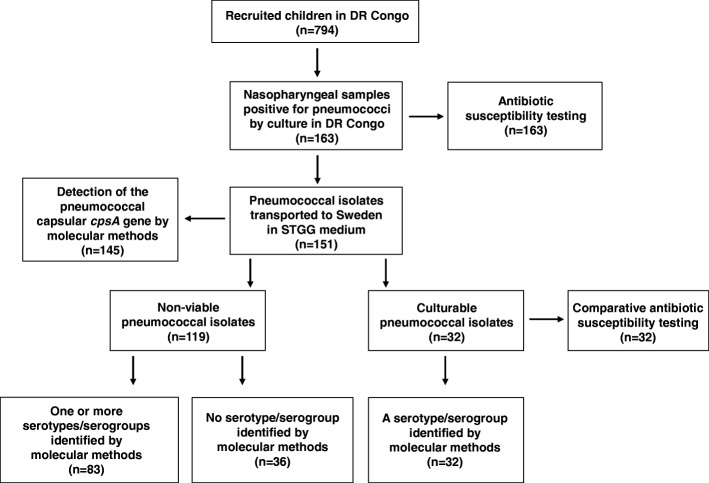
A flowchart showing the analyses performed in Bukavu, DR Congo, and in Gothenburg, Sweden, respectively, and the number of isolates included in each analysis

Out of the 163 pneumococcal strains isolated at the Clinical Laboratory at Panzi Hospital in Bukavu, 151 isolates were transported frozen in STGG medium to Gothenburg. Of these, 32 isolates could be re-cultured after storage and transport, and were tested for antibiotic susceptibility at the Department of Infectious Diseases, University of Gothenburg, Sweden, as well (Fig. 1). When the results were compared with those obtained at the laboratory in Bukavu for the same isolates, the diameter zones for all the tested antibiotic discs varied by 6 mm or less in at least 75% of the cases (Table 1). The resulting interpretation into Sensitive (S), Intermediate (I) or Resistant (R) was similar in both groups (Table 1).

**Table 1:** Comparison of disc diffusion tests on pneumococcal isolates performed in Bukavu and in Gothenburg, respectively (*n* = 32)

	Oxacillin^a^ N (%)	TMP-SMX^b^ N (%)	Erythromycin N (%)	Clindamycin N (%)	Norfloxacin^c^ N (%)	Tetracycline N (%)
Difference in disc diffusion test (mm)						
≤ 3	23 (73)	29 (91)	22 (69)	23 (73)	25 (79)	14 (44)
4–6	5 (15)	1 (3)	3 (11)	3 (11)	5 (15)	10 (31)
> 6	4 (12)	2 (6)	7 (32)	6 (18)	2 (6)	8 (25)
Difference in SIR^d^ interpretation^e^	0	0	0	0	0	0

^a^ Screening disc for beta-lactam resistance

^b^*TMP-SMX* Trimethoprim-sulphamethoxazole

^c^ Screening disc for fluoroquionolone resistance, i.e. levofloxacin and moxifloxacin

^d^*SIR* Sensitive, Intermediate, Resistant

^e^ Breakpoints used according to EUCAST 2017

The distributions of MIC values were also compared between the analyses performed in Bukavu and Gothenburg, respectively (Additional file 2). There was an even distribution of MIC values between the two sites for all of penicillin G, ampicillin and ceftriaxone. When interpreting the MIC values for penicillin G, all the isolates were categorised in the same SIR category. For ampicillin, one isolate was differently categorised into sensitive and intermediate, respectively, and the same was true in two cases for ceftriaxone. We also compared the MIC distributions between 2014 and 2015 for all the isolates tested in Bukavu and found an even distribution of the MIC values between the 2 years for ampicillin (Additional file 3). We concluded that the reproducibility was satisfactory for both the disc diffusion tests and the MIC determinations performed in Bukavu and all the results shown in the results section are therefore from the antibiotic susceptibility tests performed in Bukavu.

### Nucleic acid extraction and multiplex real-time PCR

The pneumococcal isolates were further analysed by molecular methods in Gothenburg for confirmation of species identification, and for determination of serotypes/serogroups. For those isolates that could be re-cultured after storage and transport (*n* = 32), one colony of each isolate was suspended in 1 mL of PBS prior to the extraction of nucleic acids. For unculturable isolates, 100 μL of STGG storage medium containing non-viable bacteria was diluted in 900 μL of phosphate buffered saline (PBS). DNA was extracted from 200 μL of the suspended isolates or diluted non-viable isolates using a MagNA Pure LC instrument (Roche Diagnostics, Mannheim, Germany) and the Total Nucleic acid Isolation kit (Roche Diagnostic). The extracted nucleic acids were eluted in 100 μL elution buffer. The samples were stored at − 20 °C until further analysis.

A multiplex real-time PCR, able to detect 40 different serotypes, was used according to a protocol published by Centers for Disease Control and Prevention (CDC) using previously published primers with slight modifications (preprint available at https://www.biorxiv.org/content/early/2018/09/12/415422).

The multiplex real-time PCR assays were performed using the Quant Studio 6 Flex with a 384-well system (Applied Biosystems, Carlsbad, CA). Each reaction consisted of a 20 μL reaction volume, including 4 μL of template nucleic acid, along with 1 μM of each of the forward and reverse primers, 0.85 μM of the probe, 10 μl of 2X Universal Master Mix for DNA targets (Applied Biosystems) [31] and RNAase free water. The Tecan Freedom EVO PCR setup workstation (Tecan Group Ltd., Männedorf, Switzerland) was used to prepare the PCR reactions in a 384-well plate. The PCR reaction conditions were as follows: one initial cycle at 46 °C for 2 min, followed by denaturation at 95 °C for 10 min and 45 amplification cycles of 95 °C for 15 s and 58 °C for 1 min. Each multiplex performance was evaluated using an internal control (*CpsA*) to verify the presence of pneumococcal DNA in the sample, as well as two pUC57 plasmids containing each PCR target amplicon for all serotype systems.

### Sequetyping

For eight out of the 32 pneumococcal isolates that could be re-cultured after storage and transport to Gothenburg, Sweden, and in which the multiplex PCR serotyping method was inconclusive, the serotypes/serogroups were determined using a modified Sequetyping protocol (https://www.biorxiv.org/content/early/2018/09/12/415422). Briefly, two PCR reactions were set up to amplified the whole cpsB gene. The PCR products were sent to GATC Biotech (Cologne, Germany) for purification and sequencing using the four PCR primers. The 1006 bp sequence product was matched to a reference database for determination of the serotype.

### Data management and statistical analysis

Descriptive analysis was performed using the SPSS package (version 24.0) for logistic regression to analyse the relationship between carriage and socio-demographic or medical factors. Prevalence rates and the 95% CI were calculated. Potential variables associated with pneumococcal carriage were assessed by odds ratios (OR) with 95% CI and tested by univariate analysis with the Pearson chi-square or Fisher’s exact test (*n* < 5). Associations with *p* < 0.05 were re-analysed by multivariate analysis. A *p-*value of < 0.05 was considered significant. Malnutrition was defined as the weight for age or weight for height as a Z score ≤ − 2 standard deviations, determined by ENA for smart software 2011.

## Results

### Characteristics of the included children

From seven health care centres located in the city of Bukavu, in the suburban area or in the surrounding rural area, 794 children (age range one to 60 months, median 9.0 months) were included in the study and sampled from the nasopharynx. The background health data and living conditions of the children are shown in Additional file 1.

### Socio-demographic risk factors for pneumococcal carriage

Overall, 163 (20.5%) of the children were culture positive for *S. pneumoniae* in the nasopharynx. The detection rate was associated with age but not with sex. Children aged 24–60 months had a more than three times higher rate of pneumococcal carriage that children below 6 months of age (*p-*value < 0.0001) (Table 2).

**Table 2:** Socio-demographic and medical factors related to nasopharyngeal carriage of pneumococci in children living in eastern DR Congo

Socio-demographic factors:	N carrier/N (%)	Univariate analysis		Multivariate analysis	
		OR (95% CI)	*p-*value	OR (95% CI)	*p-v*alue
Age in months					
< 6	29/302 (9.6)	1.00			
6–12	46/184 (25)	1.41 (0.85–2.36)	0.170	0.14 (0.08–0.26)	0.750
> 12–24	32/125 (26)	2.12 (1.25–3.62)	0.005	0.77 (0.44–1.36)	0.381
> 24–60	56/183 (31)	3.45 (2.19–5.44)	< 0.0001	0.90 (0.48–1.68)	< 0.0001
Sex, male	91/402 (23)	1.30 (0.92–1.84)	0.13	1.17 (0.78–1.77)	0.437
Living in rural area	98/355 (28)	2.51 (1.67–3.77)	< 0.0001	0.57 (0.33–0.97)	0.039
No of people sleeping in the same room as the child					
< 3	3/31 (9.7)	1.00			
≥ 3	74/253 (29)		0.018	1.27 (0.45–3.55)	0.644
Enclosed kitchen^a^ (*N* = 77)	43/77 (56)	6.47 (3.62–11.56)	< 0.0001	10.18 (4.93–21.02)	< 0.0001
Medical factors					
Undernutrition^b^ (*N* = 286)	83/286 (29)	2.18 (1.54–3.10)	< 0.0001	0.48 (0.32–0.73)	0.001
Current fever^c^ (*N* = 22)	14/22 (64)	5.52 (2.21–13.78)	< 0.0001	7.96 (2.38–26.58)	0.001
Previous hospitalisation (*N* = 74)	27/74 (36)	1.83 (1.04–3.25)	0.035	1.61 (0.72–3.59)	0.244
Antibiotics last month (*N* = 55)	23/55 (42)	2.32 (1.25–4.31)	0.006	2.42 (1.07–5.45)	0.033
Neonatal problems^d^ (*n* = 284)	22/51 (43.1)	2.45 (1.30–4.61)	0.004	1.27 (0.53–3.02)	0.580
PCV13 immunisation					
2 or 3 doses (*n* = 646^e^)	9/283 (3.2)	1.00			
1 dose (*n* = 773)	46/159 (29)	12.39 (5.87–26.16)	< 0.0001	30.12 (14.36–63)	< 0.0001
0 dose (n = 773)	108/331 (33)	13.47 (6.68–27.17)	< 0.0001	20.57 (9.41–44.96)	< 0.0001

^a^Enclosed kitchen = Kitchen with an open fire located inside the house directly connected to the living room and/or the bedrooms

^b^Undernutrition = weight for age or weight for height as a Z score ≤ −2 standard deviations, determined by ENA for smart software 2011

^c^Fever = 37.5–39.0 °C

^d^Neonatal problems = neonatal hospitalisation, neonatal asphyxia or neonatal resuscitation

^e^645 = the number of children that were supposed to be given ≥2 doses of PCV13 when they were older than 10 weeks or two and a half months

A higher frequency of pneumococcal carriage was observed in children living in the rural area as compared with the urban sites (28% versus 13%) and among children who lived in a house with an enclosed kitchen, i.e. with an open fire located inside the house, directly connected to the living room and/or the bedrooms, and these associations remained significant in multivariate analysis (Table 2). The type of stove and fuel for cooking did not correlate with carriage. Nor were there any associations between pneumococcal carriage and the number of rooms, having siblings, parents smoking tobacco, type of building material in the walls or the roof of the house, or having an animal in the household (Additional file 4).

### Medical risk factors

Immunisation with PCV13 was strongly associated with lower rates of pneumococcal carriage, which was observed in only 3% of children who had received two or three doses of PCV13 as compared with approximately 30% of the unvaccinated children (*p* < 0.0001) (Table 2). Malnourished children, children with current fever and those who had had recent antibiotic treatment were more commonly colonised with pneumococci than children without these factors (*p* < 0.05). In contrast, neonatal problems, asthma, a recent history of malaria or gastroenteritis, or immunisation against measles, tuberculosis or *Hemophilus influenzae* type B were not associated with carriage, nor were symptoms of upper respiratory airway infection, such as a runny nose or cough (Table 2 and Additional file 5).

Taken together, age, living in a household with an enclosed kitchen, living in a rural area, undernutrition, current fever and antibiotic treatment during the last month were significantly associated with a higher, and vaccination with PCV13 with a lower, frequency of pneumococcal detection (Table 2).

### Antimicrobial susceptibility of S. pneumoniae isolates

**Fig. 2:**
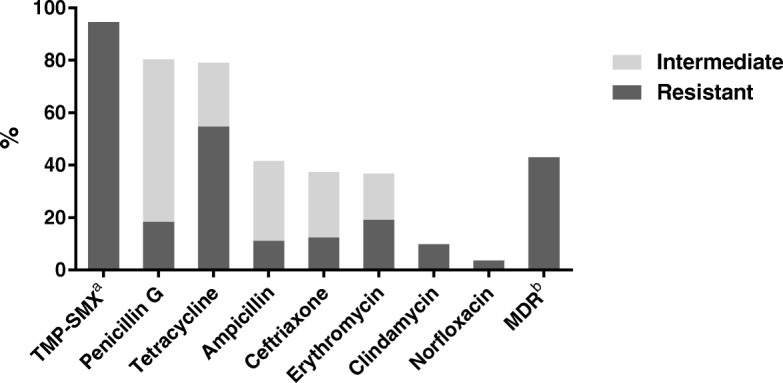
The antimicrobial susceptibility pattern was determined in Bukavu, DR Congo for 163 pneumococcal strains isolated from healthy Congolese children. Disc diffusion tests were performed to detect reduced susceptibility to oxacillin, trimethoprim-sulfamethoxazole (TMP-SMX), tetracycline, erythromycin, clindamycin or norfloxacin (screening for fluoroquinolone resistance, i.e. levofloxacin and moxifloxacin). For oxacillin non-susceptible isolates, the minimal inhibitory concentration (MIC) was determined for penicillin G, ampicillin and ceftriaxone. ^a^TMP-SMX = trimethoprim-sulfamethoxazole, ^b^MDR = multi-drug resistant, i.e. non-susceptible to ≥3 classes of antibiotics including the beta-lactams

The antimicrobial susceptibility pattern was determined at the Clinical Laboratory, Panzi Hospital, Bukavu, for the 163 pneumococcal strains that were isolated from the children (Fig. 2). Using disc diffusion tests, 145 (89%) of the isolates were shown to be non-susceptible to oxacillin and they were therefore regarded as resistant to phenoxymethylpenicillin. These 145 strains were further tested by MIC determination against penicillin G and 101 (62%) strains were categorised as intermediate (MIC 0.06–2 mg/L), while 30 (18%) were resistant (MIC > 2 mg/L). Taken together, 131/163 (80%) of the strains showed reduced susceptibility to benzylpenicillin, as confirmed by MIC determination (Fig. 2). Sixty-eight isolates (42%) had reduced susceptibility to ampicillin, of which 18 were resistant (MIC > 2 mg/L), and 61 isolates (37%) had reduced susceptibility to ceftriaxone (Fig. 2). High rates of non-susceptibility were also found for tetracycline and as many as 94% of the isolates were resistant to trimethoprim-sulphamethoxazole (TMP-SMX), also known as co-trimoxazole (Fig. 2). Notably, 70 (43%) of the pneumococci were multidrug resistant (non-susceptible to ≥3 classes of antimicrobial agents, including the beta-lactams).

### Serotype distribution

The serotypes or serogroups of the isolated pneumococci were determined by multiplex real-time PCR or by the modified Sequetyping protocol, both performed in Gothenburg, Sweden. Among the 32 living isolates the most common serotype was 19F (*n* = 11), followed by 11A/D (*n* = 5) and 35B/35C (n = 5). The pneumococcal capsular *cpsA* gene was detected in all viable isolates, confirming their species identification.

**Fig. 3:**
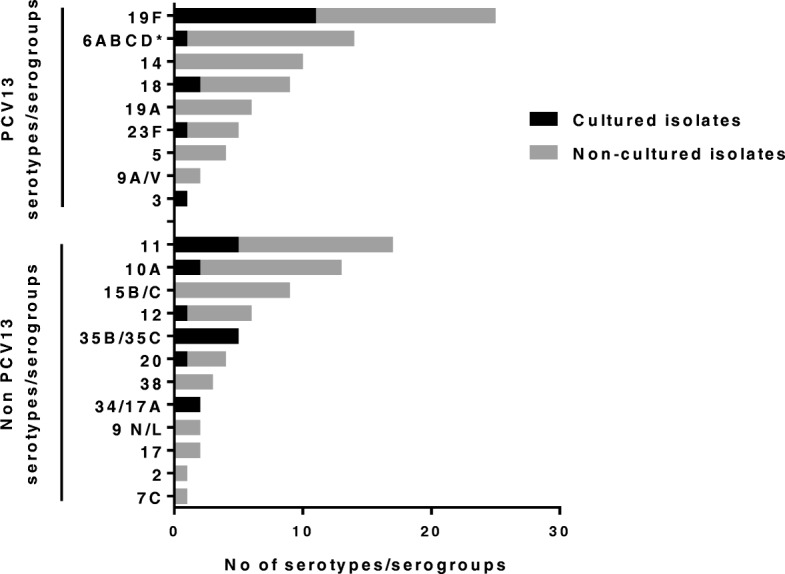
The combined results of the 141 serotypes/serogroups identified by multiplex PCR or Sequetyping in the cultured, living pneumococcal isolates (*n* = 32) and by multiplex PCR in the non-viable pneumococcal isolates stored in tubes containing bacteria and STGG medium (*n* = 83). In 21 of these tubes containing non-viable bacteria, two or more serotypes/serogroups could be detected (two serotypes/serogroups in 17 cases; three serotypes/groups in three cases and four serotypes/groups in one case). * One culturable isolate could be determined by Sequetyping as 6B

The serotypes/serogroups of the pneumococci that could not be re-cultured were determined by multiplex PCR after isolation of genomic material from the non-viable isolates in the STGG storage medium (*n* = 119). In 62/119 cases (52%), one serotype/serogroup could be identified, whereas in 21 cases more than one serotype/serogroup was detected. Of these, two serotypes/groups were determined in 17 cases, three serotypes/groups in three cases, while one tube contained four serotypes/groups. In 36/119 cases (30%), no serotype or group could be determined by multiplex real-time PCR. The combined results for all 141serotypes/serogroups that were identified in the viable and non-viable pneumococcal isolates are shown in Fig. 3.

Hence, out of the 141 identified serotypes/serogroups, 76 (54%) belonged to a serotype/serogroup included in PCV13. However, these 76 included 13 pneumococcal strains that could not be distinguished between 6A, 6B, 6C and 6D, of which only 6A and 6B are included in PCV13. Two further strains could not be separated between 9A and 9 V, of which only 9 V is included. Sixty-five (46%) of the identified serotypes/serogroups could, however, be categorised as non-PCV13-containing types/groups. Thus, the proportion of identified serotypes/serogroups belonging to PCV13 was similar to those not belonging to the vaccine. In the nine children who had received two or three doses of PCV13, vaccine serotypes/groups (*n* = 9) were as commonly detected as were serotypes/groups not included in the vaccine (*n* = 7). There was no significant difference in the distribution of penicillin non-susceptibility between strains whose serotypes/serogroups are included in the PCV13 compared to strains with non-vaccine serotypes/groups (Additional file 6).

In 113/119 non-viable isolates, the pneumococcal capsule gene, *CpsA*, could be detected in the STGG medium.

In the 36 samples, in which no serotype/serogroup could be identified using the multiplex real-time PCR method, the capsule gene, *CpsA*, was detected in 34 cases, verifying the presence of pneumococcal genomic material in the samples, and excluding a complete pneumococcal degradation during transport and storage.

## Discussion

This is the first study to report the prevalence, serotype distribution and antimicrobial susceptibility of *Streptococcus pneumoniae* carried by healthy children in DR Congo.

By using culture for pneumococcal detection, we found that the prevalence of nasopharyngeal carriage was 21%. Generally higher levels of carriage have been reported from several other African countries, but there is also a great deal of variation between different regions [21, 32–35].

Differences in carriage rates can be due to geographical and regional variations, or to methodological differences. Limitations of the culture procedures in the present study include use of human blood in the agar plates. The pneumococci were initially identified in Bukavu, DR Congo, using the optochin test, which is not recommended as single test for *S. pneumoniae* identification [36]*.* However, molecular methods performed in Sweden confirmed the species identification in all but two isolates, either by detection of the pneumococcal capsular gene *CpsA*, or by serotype/serogroup determination. The low rate of positive culture in Sweden after storage and transport could be due to activation of the pneumococcal enzyme autolysin, LytA, which causes the bacterium to lyse and die. If activation of the autolysin also had an impact on the initial cultures performed in Bukavu can only be speculated upon. Apart from potential methodological limitations, our relatively low detection rate could indeed reflect an early effect of the newly introduced pneumococcal conjugate vaccine. To our knowledge, only a few studies have determined the carrier rate of pneumococci among sub-Saharan children after the introduction of the pneumococcal conjugate vaccine [13, 16].

Our study showed that living in a rural area was associated with a higher rate of pneumococcal carriage than in an urban area, in agreement with other studies showing the socio-economic and geographical disparity of pneumococcal carriage [37, 38]. We also found a strong association between pneumococcal carriage and living in a house with an enclosed kitchen, i.e. with an open fire located inside the house, similar to what has been reported before [39]. Many studies have shown that an increased risk of pneumonia and other lower respiratory infections correlates with household air pollution and poverty, but few have studied the association with pneumococcal carriage. Hussey et al. have reported that air pollution alters pneumococcal biofilms, antibiotic tolerance and colonisation [40]. Here, we also confirm the relationship between malnutrition and pneumococcal carriage, as described before [37, 41, 42].

We found an increased prevalence of pneumococci with age, in agreement with the results from Niger [33], while other studies in Africa showed the contrary [38, 43, 44]. One explanation of this could be that the sampling started a few months after the introduction of a PCV13 vaccine programme in DR Congo. Since the children are given vaccine doses when they are six, ten and 14 weeks old, without a catch-up programme, most of the under 2 year olds were vaccinated, while most of the children older than 2 years of age were not. The majority of the children under 6 months of age had already received three doses of PCV13.

Although the vaccination status could not be confirmed by checking the immunisation child cards in all cases, but instead relied on self-reports of the caretakers, we found that PCV13 immunisation was highly protective against pneumococcal carriage. Only 3% of the children that had received two or three doses of PCV13 carried pneumococci, compared with approximately 30% in those that were unvaccinated or had only been given one dose. Among the 141 serotypes/serogroups that were identified, approximately half belonged to a serotype/serogroup included in PCV13, which is similar to other studies [38, 44–47]. Since 24% of the isolates could not be identified to serotype/serogroup and the fact that the multiplex PCR was developed mainly to cover PCV-containing serotypes, it is possible that the number of non-vaccine serotypes/serogroups could be even higher. In Kenya, the prevalence of vaccine serotypes was reduced from 34 to 13% after the introduction of PCV10 [13]. The predominant serotype circulating in the eastern part of DR Congo was found to be the vaccine-type 19F, corroborating the results from Mozambique [48] and Ghana [22]. We detected equally numbers of vaccine- and non-vaccine-serotypes/groups in the nine children who had received two or more doses of PCV13. The distribution of penicillin non-susceptible strains was also similar between the identified vaccine- and non-vaccine serotypes/groups, indicating a limited effect of PCV13 on the carriage of antibiotic resistant strains in the area shortly after introduction of the vaccine.

The relatively high prevalence of serotype 11A/D and 35B/35C, which are not included in the PCV13 vaccine, among the living isolates was unexpected and has not been reported in other African studies [21, 38, 49]. As there are no studies of pneumococcal carriage in DR Congo prior to the introduction of PCV13, no evaluation of serotype replacement can be performed.

In some of the samples containing non-viable pneumococci, we unexpectedly detected more than one serotype/serogroup, although the STGG medium was supposed to contain only one pure cultured pneumococcal isolate. This might reflect the difficulties to visually separate different pneumococcal strains according to their colony morphology on the agar plate, and also confirms other observations that children often carry more than one pneumococcal strain in nasopharynx [50, 51].

We found an association between antibiotic treatment within 1 month prior to sampling and pneumococcal carriage among the children, similar to a study from Iran [39], but contrary to the results from Kenya [52] and Niger [33]. This finding indicates the carriage of pneumococci resistant to antibiotics used in the area and we were in fact able to show alarmingly high resistance rates to the antibiotics commonly used in the eastern part of DR Congo. Amoxicillin (or intravenous ampicillin in severe cases) is recommended by the WHO as the first-line treatment for pneumonia. We found that 42% of the isolated pneumococci had reduced susceptibility to ampicillin, while the rate of non-susceptibility to benzylpenicillin was 80%. High rates of pneumococcal non-susceptibility to ampicillin and/or penicillin have been reported in other sub-Saharan countries [53, 54], but lower rates have also been observed [21, 22, 33]. There is some reported high resistance to TMP-SMX, as we found in this study (94%) [44, 55]. One of the rare post-PCV studies in sub-Saharan Africa reported a limited impact on antibiotic resistance [56]. TMP-SMX or co-trimoxazole and penicillin are the two most available and accessible antibiotics in DR Congo [57]. Self-medication is fairly common in the country, due to inadequate access to formal health care and the wide availability of antibiotics without prescription [57].

Due to the absence of a national antibiotic use policy in DR Congo, non-governmental organisations have introduced their guidelines on empirical antibiotic treatment recommendations, without having enough data on local antibiotic resistance rates. Until recently, co-trimoxazole was recommended as empirical treatment for acute lower respiratory infections in DR Congo, instead of amoxicillin, as recommended by the WHO, and the use of this antibiotic is still widespread in the country. In addition, in many cases, HIV-positive children are likely to be administered TMP-SMX as prophylaxis for *Pneumocystis jirovecii* infections, which might contribute to the development of TMP-SMX-resistant pneumococci.

In DR Congo, the cephalosporin ceftriaxone is the most commonly used antimicrobial drug for severe pneumococcal infections like meningitis. Here, we demonstrate a higher level of resistance (37%), compared with previous studies from Botswana [58] and Tanzania [21].

Moreover, many isolated pneumococcal strains also had reduced susceptibility to erythromycin, tetracycline and clindamycin. Notably, a large proportion of pneumococcal isolates (43%) were multi-drug resistant, i.e. non-susceptible to ≥3 classes of antimicrobial agents, in contrast to the situation in other countries close to DR Congo [21, 44, 53].

The high level of antimicrobial resistance found in our study can be explained by the absence of regulation for the use of antibiotics and no national guidelines for the management of frequent diseases in DR Congo. Moreover, there is an urgent need for microbiological competence and knowledge, as well as well-equipped laboratories, capable of clinical diagnostics and antibiotic resistance surveillance.

## Conclusions

To conclude, this study was performed on healthy children below 5 years of age in the eastern part of DR Congo after the introduction of PCV13. Living in rural areas, having an enclosed kitchen with an open fire and undernutrition correlated with higher pneumococcal carriage and PCV13 vaccination with a lower carriage rate. Moreover, it highlights an alarmingly high level of reduced susceptibility to commonly used antibiotics, especially ampicillin and ceftriaxone, among the isolated pneumococcal strains. This underlines the need for new antibiotic treatment guidelines, as well as necessitating local and national antibiotic resistance surveillance programmes.

## Additional files

Additional file 1: Socio-demographic factors of the children as reported by the parents (PDF 189 kb)

Additional file 2: Susceptibility testing of Penicillin G, Ampicillin and Ceftriaxone performed in Bukavu, D.R. Congo and in Gothenburg, Sweden. To compare the antibiotic susceptibility tests performed in Bukavu, DR Congo, with those performed in Gothenburg, Sweden, the minimal inhibitory concentration (MIC) was determined for penicillin G, ampicillin and ceftriaxone in 32 pneumococcal isolates at both sites. (PPTX 53 kb)

Additional file 3: The distribution of MIC values for penicillin G, ampicillin and ceftriaxone, respectively, obtained in Bukavu during 2014 and 2015. (PPTX 49 kb)

Additional file 4: Socio-demographic factors of the children in relation to nasopharyngeal pneumococcal carriage. (PDF 91 kb)

Additional file 5: Medical factors of the children in relation to pneumococcal carriage. (PDF 167 kb)

Additional file 6: Distribution of penicillin non-susceptibility (intermediate or resistant) among pneumococcal isolates in which a serotype/serogroup included in the PCV13 was detected, and among isolates in which a serotype/group not included in the vaccine was determined (PPTX 55 kb)

## Abbreviations

CARe: Center for Antibiotic Resistance Research Gothenburg
CDC: Centers for Disease Control and Prevention
CI: Confidence interval
DR Congo: Democratic Republic of the Congo
ENA: Emergency Nutrition Assessment
EUCAST: European committee on antimicrobial susceptibility testing
HIV: Human immunodeficiency virus
MIC: Minimum inhibitory concentration
MSF: Médecins Sans Frontières
OR: Odds ratio
PCV: Pneumococcal conjugate vaccine
PCV10: 10 valent pneumococcal conjugate vaccine
PCV13: 13 valent pneumococcal conjugate vaccine
PCV7: 7-valent pneumococcal conjugate vaccine
TMP-SMX: Trimethoprim-sulphamethoxazole
WHO: World Health Organisation
