# Supplementary material for: High rate of antibiotic resistance among pneumococci carried by healthy children in the eastern part of the Democratic Republic of the Congo
Source: BMC Pediatr. 2018 Nov 19;18:361. doi: 10.1186/s12887-018-1332-3 (PMC6241069; doi:10.1186/s12887-018-1332-3)
Supplement: Supplementary file 1 — Socio-demographic factors of the children as reported by the parents (PDF 189 kb) [file 12887_2018_1332_MOESM1_ESM.pdf]

Additional File 1:
Socio-demographic factors as reported by the parents

Characteristics		N	%
**Health centre (n=794)**	Kadutu (urban)	99	12
	Kaziba (rural)	109	14
	Malkiya wa Amani (urban)	100	13
	Muhanzi (urban)	106	13
	Muku (rural)	119	15
	Nyantende (rural)	126	16
	Panzi (suburban)	135	17
**Location of residence (n=794)**	Urban	304	38.3
	Rural	355	44.7
	Suburban	135	17.0
**Sex (n=794)**	Female	392	49.4
**Age (months) (n=794)**	< 6	302	38.0
	6 – 12	184	23.2
	> 12 – 24	125	15.7

	> 24 – 60	183	23.1
**Nutritional status (n=794)**	Undernutrition[1]	286	36.0
**Mother's occupation (n=284)**	Agriculture	197	69.4
	Self-employed	61	21.4
	Employed	25	8.8
	Unknown	1	0.4
**Father's occupation (n=284)**	Agriculture	113	39.8
	Self-employed	109	38.4
	Employed	34	12.0
	Unknown	21	7.4
	Other jobs	7	2.4
**Mother's education (n=284)**	No education	149	52.4
	Primary school	65	22.9
	Secondary school	65	22.9
	Occupational studies	4	1.4
	University	1	0.4
**Father's education (n=284)**	No education	67	23.6
	Primary school	98	34.5
	Secondary school	85	30.0

	Occupational studies	23	8.1
	University	11	3.9
**Number of rooms in the house (n=284)**	0-1	91	32.0
	2-3	160	56.4
	> 3	33	11.6
**People living in the house (n=284)**	0-4	30	10.6
	5-7	133	46.8
	> 7	121	42.6
**People sleeping in the same room as the child (n=284)**	1 - 2	31	10.9
	3 – 4	218	76.8
	> 4	35	12.3
**Children < 5 years of age living in the house (n=284)**	0-2	47	16.6
	3-4	221	77.8
	> 4	16	5.6
**Siblings (n=284)**	0-2	80	28.2
	3-4	103	36.2

	>4	101	35.6
**Having animals in the house (n=284)**		32	11.2
**Partly breastfeeding (months) (n=284)**	< 6	7	2.5
	7-12	38	13.4
	12-24	165	58.1
	> 24	74	26.0
**Kitchen (n=284)**	Enclosed in house	77	27.1
**Most important fuel for cooking (n=284)**	Wood	180	63.4
	Charcoal	94	33.1
	Electricity	10	3.5
**Most used stove for cooking (n=284)**	Free stones	179	63.0
	Charcoal stove	94	33.1
	Grid electric	11	3.9
**Parental tobacco smoking (n=284)**		11	3.9
**Immunisation BCG[2] (n = 794)**		783	98.6
**Immunisation HiB[3] (n = 772)**	None	55	71.2

	1 dose	77	10.0
	2 or 3 doses	641	83.0
**Immunisation PCV13**	None (n= 772)	331	42.9
	1 dose (n = 772)	159	20.6
	2 or 3 doses (n =645[4])	283	43.9
**Immunisation measles (n = 431)**		343	79.6
**Ongoing symptoms (n=284)**	Fever[5]	22	7.7
	Chills	21	7.4
	Cough	88	31.0
	Runny nose	25	8.8
**Past history of disease (n=284)**	Malaria	44	15.5
	Gastroenteritis	41	14.4
	Neonatal problems	51	18.0
	Asthma/bronchiolitis	14	4.9
	Other diseases[6]	31	10.9
	HIV	0	0
	Tuberculosis	0	0
	Heart disease	0	0
**Previous hospitalisation (n=284)**		74	26.0

**Use of antibiotics last month (n=284)**	55	19.4

[1] Undernutrition defined as weight for age or weight for height as a Z score ≤ -2 standard deviations, determined by ENA for smart software 2011 [26],

[2] BCG =Bacillus Calmette–Guérin vaccine, [3] HiB=*Haemophilus influenzae* type B vaccine,

[4] 645 = the number of children that were supposed to be given ≥ 2 doses of PCV13 when they were older than 10 weeks or two and a half months,

[5] Fever = 37.5-39.0°C),

[6] Other diseases = genetic disorders (sickle cell disease n= 2, Down syndrome n=1), cerebral palsy n=3
