# Supplementary material for: High rate of antibiotic resistance among pneumococci carried by healthy children in the eastern part of the Democratic Republic of the Congo
Source: BMC Pediatr. 2018 Nov 19;18:361. doi: 10.1186/s12887-018-1332-3 (PMC6241069; doi:10.1186/s12887-018-1332-3)
Supplement: Supplementary file 2 — Susceptibility testing of Penicillin G, Ampicillin and Ceftriaxone performed in Bukavu, D.R. Congo and in Gothenburg, Sweden. To compare the antibiotic susceptibility tests performed in Bukavu, DR Congo, with those performed in Gothenburg, Sweden, the minimal inhibitory concentration (MIC) was determined for penicillin G, ampicillin and ceftriaxone in 32 pneumococcal isolates at both sites. (PPTX 53 kb) [file 12887_2018_1332_MOESM2_ESM.pptx]

## Slide 1
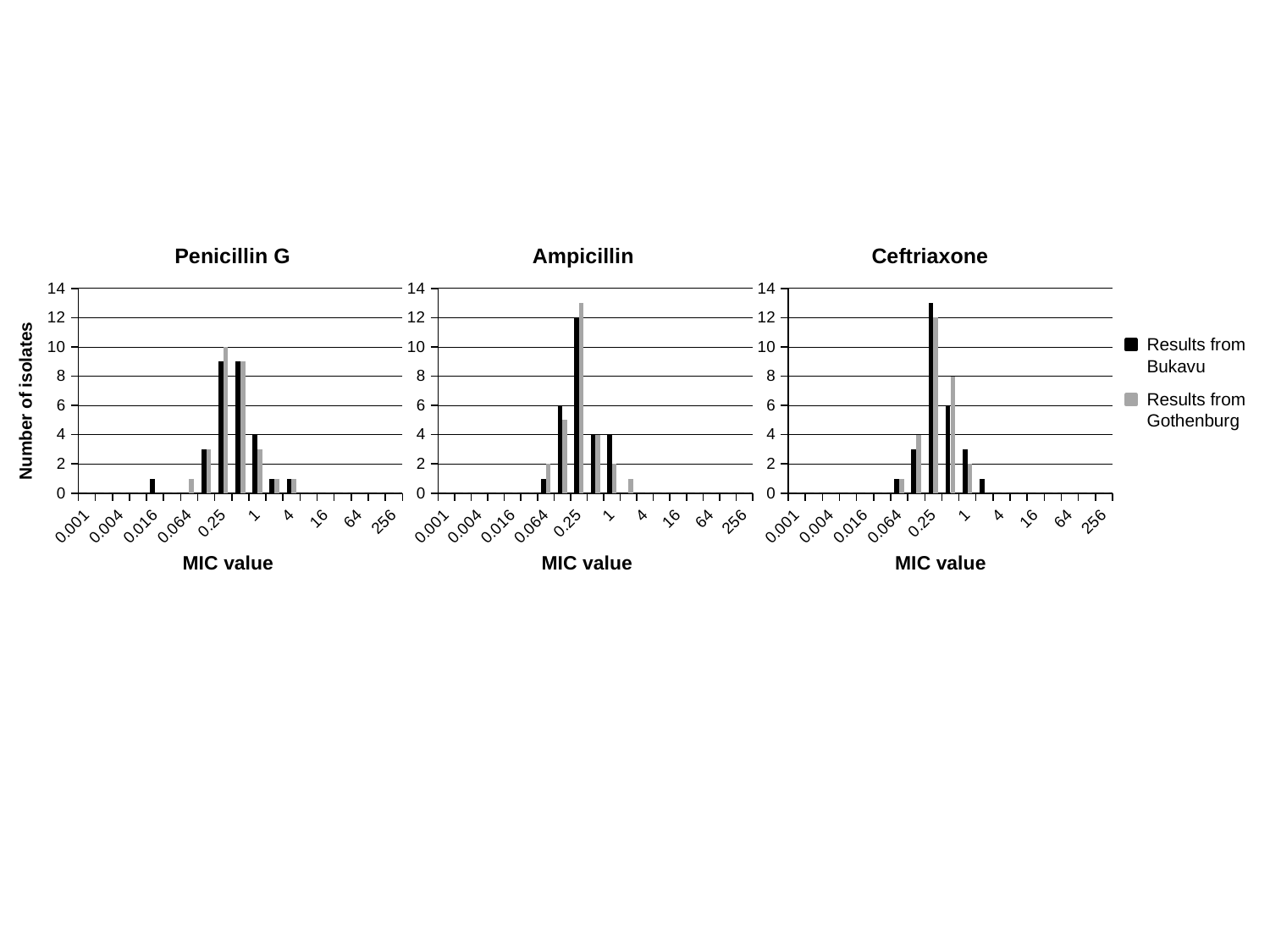

Ceftriaxone
Penicillin G
Ampicillin
### Chart
| Category | Results from Bukavu | Results from Gothenburg |
|---|---|---|
| 0.001 | 0.0 | 0.0 |
| 0.002 | 0.0 | 0.0 |
| 0.004 | 0.0 | 0.0 |
| 0.008 | 0.0 | 0.0 |
| 0.016 | 1.0 | 0.0 |
| 0.032 | 0.0 | 0.0 |
| 0.064 | 0.0 | 1.0 |
| 0.125 | 3.0 | 3.0 |
| 0.25 | 9.0 | 10.0 |
| 0.5 | 9.0 | 9.0 |
| 1 | 4.0 | 3.0 |
| 2 | 1.0 | 1.0 |
| 4 | 1.0 | 1.0 |
| 8 | 0.0 | 0.0 |
| 16 | 0.0 | 0.0 |
| 32 | 0.0 | 0.0 |
| 64 | 0.0 | 0.0 |
| 128 | 0.0 | 0.0 |
| 256 | 0.0 | 0.0 |
### Chart
| Category | MICAPadjustedBkv | MICAPadjustedGtbg |
|---|---|---|
| 0.001 | 0.0 | 0.0 |
| 0.002 | 0.0 | 0.0 |
| 0.004 | 0.0 | 0.0 |
| 0.008 | 0.0 | 0.0 |
| 0.016 | 0.0 | 0.0 |
| 0.032 | 0.0 | 0.0 |
| 0.064 | 1.0 | 2.0 |
| 0.125 | 6.0 | 5.0 |
| 0.25 | 12.0 | 13.0 |
| 0.5 | 4.0 | 4.0 |
| 1 | 4.0 | 2.0 |
| 2 | 0.0 | 1.0 |
| 4 | 0.0 | 0.0 |
| 8 | 0.0 | 0.0 |
| 16 | 0.0 | 0.0 |
| 32 | 0.0 | 0.0 |
| 64 | 0.0 | 0.0 |
| 128 | 0.0 | 0.0 |
| 256 | 0.0 | 0.0 |
### Chart
| Category | Results from Bukavu | Results from Gothenburg |
|---|---|---|
| 0.001 | 0.0 | 0.0 |
| 0.002 | 0.0 | 0.0 |
| 0.004 | 0.0 | 0.0 |
| 0.008 | 0.0 | 0.0 |
| 0.016 | 0.0 | 0.0 |
| 0.032 | 0.0 | 0.0 |
| 0.064 | 1.0 | 1.0 |
| 0.125 | 3.0 | 4.0 |
| 0.25 | 13.0 | 12.0 |
| 0.5 | 6.0 | 8.0 |
| 1 | 3.0 | 2.0 |
| 2 | 1.0 | 0.0 |
| 4 | 0.0 | 0.0 |
| 8 | 0.0 | 0.0 |
| 16 | 0.0 | 0.0 |
| 32 | 0.0 | 0.0 |
| 64 | 0.0 | 0.0 |
| 128 | 0.0 | 0.0 |
| 256 | 0.0 | 0.0 |Results from
Bukavu
Results from
Gothenburg
Number of isolates
MIC value
MIC value
MIC value
