# Supplementary figures and images for: High rate of antibiotic resistance among pneumococci carried by healthy children in the eastern part of the Democratic Republic of the Congo
Source: BMC Pediatr. 2018 Nov 19;18:361. doi: 10.1186/s12887-018-1332-3 (PMC6241069; doi:10.1186/s12887-018-1332-3)

## Slide 1
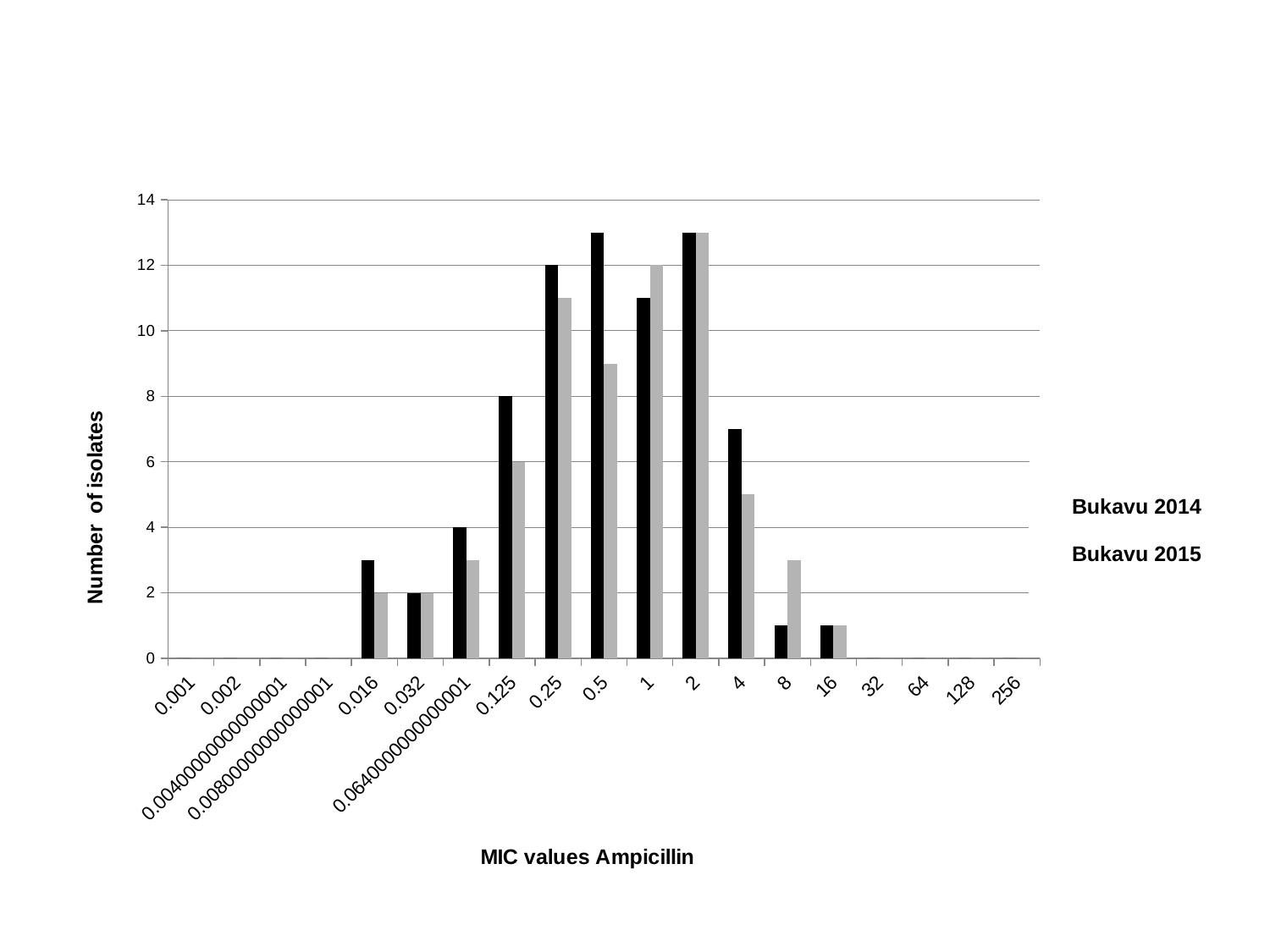

### Chart
| Category | MICAP2014 | MICAP2015 |
|---|---|---|
| 0.001 | 0.0 | 0.0 |
| 0.002 | 0.0 | 0.0 |
| 0.00400000000000001 | 0.0 | 0.0 |
| 0.00800000000000001 | 0.0 | 0.0 |
| 0.016 | 3.0 | 2.0 |
| 0.032 | 2.0 | 2.0 |
| 0.0640000000000001 | 4.0 | 3.0 |
| 0.125 | 8.0 | 6.0 |
| 0.25 | 12.0 | 11.0 |
| 0.5 | 13.0 | 9.0 |
| 1.0 | 11.0 | 12.0 |
| 2.0 | 13.0 | 13.0 |
| 4.0 | 7.0 | 5.0 |
| 8.0 | 1.0 | 3.0 |
| 16.0 | 1.0 | 1.0 |
| 32.0 | 0.0 | 0.0 |
| 64.0 | 0.0 | 0.0 |
| 128.0 | 0.0 | 0.0 |
| 256.0 | 0.0 | 0.0 |
Bukavu 2014
Bukavu 2015

Supplement: Supplementary file 3 — The distribution of MIC values for penicillin G, ampicillin and ceftriaxone, respectively, obtained in Bukavu during 2014 and 2015. (PPTX 49 kb) [file 12887_2018_1332_MOESM3_ESM.pptx]

## Slide 1
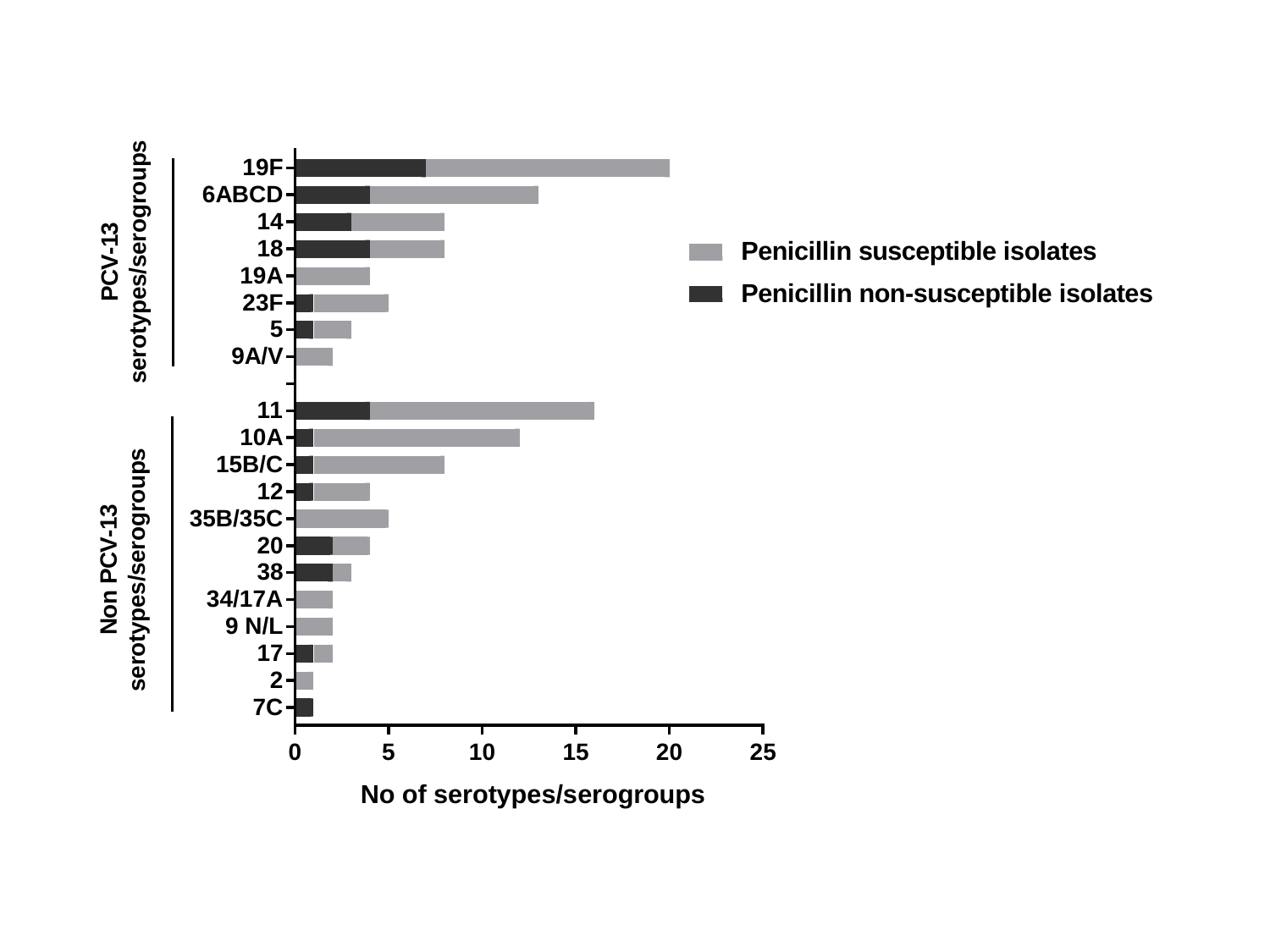

Supplement: Supplementary file 6 — Distribution of penicillin non-susceptibility (intermediate or resistant) among pneumococcal isolates in which a serotype/serogroup included in the PCV13 was detected, and among isolates in which a serotype/group not included in the vaccine was determined (PPTX 55 kb) [file 12887_2018_1332_MOESM6_ESM.pptx]
