# Supplementary material for: High rate of antibiotic resistance among pneumococci carried by healthy children in the eastern part of the Democratic Republic of the Congo
Source: BMC Pediatr. 2018 Nov 19;18:361. doi: 10.1186/s12887-018-1332-3 (PMC6241069; doi:10.1186/s12887-018-1332-3)
Supplement: Supplementary file 4 — Socio-demographic factors of the children in relation to nasopharyngeal pneumococcal carriage. (PDF 91 kb) [file 12887_2018_1332_MOESM4_ESM.pdf]

Additional File 4

Socio-demographic factors of the children in relation to nasopharyngeal pneumococcal carriage

Socio-demographic factors		N (%)	Pneumococcal carriage N (%)	OR (95% CI)	*p*-value
**Sex (n=794)**	**Female**	392(49)	72(18)	1.00	
	**Male**	402(51)	91(23)	1.30(0.92-1.84)	0.13
**Age (n=794)**	**< 6 months**	302(38)	29(9.6)	1.00	
	**6 – 12 Months**	184(23)	46(25)	1.41(0.85-2.36)	0.170
	**> 12 – 24 months**	125(16)	32(26)	2.12(1.25-3.62)	0.005
	**> 24 – 60 months**	183(23)	56(31)	3.45(2.19-5.44)	<0.0001
**Location of residence (n=794)**	**Urban**	304(38)	40(13)	1.00	
	**Rural**	355(45)	98(28)	2.51(1.67-3.77)	<0.0001
	**Sub- Urban**	135(17)	25(19)	1.50(0.86-2.59)	0.146
**Number of rooms in the house (n=284)**	**0-1 room**	91(32)	19(21)	1.00	
	**2-3 rooms**	160(56)	47(29)	1.57(0.85-2.89)	0.141
	**> 3rooms**	33(12)	11(33)	1.89(0.78-4.58)	0.15
**People living in the house (n=284)**	**0-4**	30(11)	9(29)	1.00	
	**5-7**	133(47)	40(30)	1.00(0.42-2.38)	0.998

	**> 7**	121(43)	28(23)	0.70(0.28-1.70)	0.430
**People sleeping in the same room as the child[2] (n=284)**	**1 – 2**	31(11)	3(9.7)	1.00	
	**> 3**	253(89)	74(29)		0.018
**Children < 5 years of age living in the house (n=284)**	**0-2**	47(17)	13(28)	1.00	
	**3-4**	221(78)	58(26)	0.93(0.45-1.88)	0.09
	**> 4**	16(5.6)	6(38)	1.56(0.47-5.19)	0.46
**Siblings (n=284)**	**0-2**	80(28)	27(34)		0.197
	**3-4**	103(36)	29(28)		0.430
	**5-6**	79(28)	17(22)		0.990
	**> 6**	22(7.7)	4(18)	1.00	
**Having animal in the house (n=284)**		32(11)	7(22)	0.47(0.30-1.76)	0.48
**Breast feeding (n=284)**	**< 6 Months**	7(2.5)	1(14)	1.00	
	**7-12 Months**	38(13)	11(29)	2.44(0.26-22.73)	0.43
	**12-24 months**	166(58)	44(27)	2.16(0.25-18.48)	0.48
	**> 24 months**	74(26)	21(28)	2.37(0.26-20.95)	0.43
**Kitchen (n=284)**	**Enclose Kitchen[1]**	77(27)	43(56)	6.47(3.62-11.56)	<0.0001
**Most important fuel for cooking (n=284)**	**Wood**	180(63)	50(28)		0.730

	**Charcoal**	94(33)	26(28)		0.725
	**Electricity**	10(4.0)	2(20)	1.00	
**Stove most often used for cooking (n=284)**	**Free stones**	179(63)	49(27)		0.730
	**Shield stove**	94(33)	26(28)		0.723
	**Grid Electric**	11(3.9)	2(18)	1.00	
**Parental tobacco smoking (n=284)**		11(3.9)	2(18)		0.523

[1]Enclosed kitchen= Kitchen with an open fire located inside the house directly connected to the living room and/or the bedrooms

[2]People sleeping in the same room as the child: > 5 years child Sleeping in the same room with the child
