# Supplementary material for: High rate of antibiotic resistance among pneumococci carried by healthy children in the eastern part of the Democratic Republic of the Congo
Source: BMC Pediatr. 2018 Nov 19;18:361. doi: 10.1186/s12887-018-1332-3 (PMC6241069; doi:10.1186/s12887-018-1332-3)
Supplement: Supplementary file 5 — Medical factors of the children in relation to pneumococcal carriage. (PDF 167 kb) [file 12887_2018_1332_MOESM5_ESM.pdf]

Additional File 5

Medical factors of the children in relation to pneumococcal carriage

Medical Factors		N (%)	Pneumococcal carriage N (%)	OR (95% CI)	*p*-Value
**Nutritional status (n=794)**	**Under nutrition**[1]	286(36)	83(29)	2.18(1.54-3.10)	<0.0001
**Immunization BCG**[2] **(n = 794)**	**BCG**	783(99)	161(21)	Fisher test	0.998
**Immunization HIB**[3] **(n = 773)**	**0 dose (n = 773)**	55(7.1)	5(9.1)	1.00	
	**1 dose (n =773)**	77(10)	15(20)	2.41(0.82-7.11)	0.108
	**2 or 3 doses (n = 773)**	641(83)	148(23)	3.01(1.75-7.66)	0.021
**Immunization PCV13 (n = 773)**	**2 or 3 doses (n =646**[4]**)**	283(44)	9(3.2)	1.00	
	**1 dose (n = 773)**	159(21)	46(29)	12.39(5.87-26.16)	<0.0001
	**0 dose (n= 773)**	331(43)	108(33)	13.47(6.68-27.17)	<0.0001
**Measles Immunization (n = 431)**	**Measles (n = 431)**	343(80)	84(24)	1.68(0.90-3.13)	0.1006
**Ongoing symptoms (n=284)**	**Fever**[5] **(n=284)**	22(7.7)	14(64)	5.52(2.21-13.78)	<0.0001
	**Chills (n=284)**	21(7.4)	6(29)	1.082(0.40-2.89)	0.87
	**Cough (n=284)**	87(31)	23(26)	0.95(0.53-1.68)	0.86
	**Runny nose (n=284)**	25(8.8)	9(36)	1.58(0.67-3.74)	0.29

**Past history of diseases (n=284)**	**Malaria (n=284)**	41(14)	11(27)	0.98(0.46-2.08)	0.98
	**Gastroenteritis (n=284)**	43(15)	13(30)	1.19(0.58-2.44)	0.617
	**Neonatal problems[6] (n=284)**	51(18)	22(43)	2.45(1.30-4.61)	0.004
	**Asthma/Bronchiolitis (n=284)**	14	4(29)	Fisher test	0.998
	**Others diseases[7] (n=284)**	31(11)	9(29)	1.11(0.48-2.53)	0.789
	**HIV (n=284)**	00(0.0)	0.0		
	**Tuberculosis (n=284)**	00(0.0)	0.0		
	**Heart disease (n=284)**	00(0.0)	0.0		
**Posterior Hospitalization (n=284)**		74(26)	27(36)	1.83(1.04-3.25)	0.035
**Use of antibiotic last month (n=284)**	**One month before the sampling**	55(19)	23(42)	2.32(1.25-4.31)	0.006

[1] Undernutrition defined as

Weight for age or weight for height as a Z score ≤ -2 standard deviations, determined by ENA for smart software 2011

[2] BCG =Bacillus Calmette–Guérin vaccine

[3] HiB=*Haemophilus influenzae* type B vaccine

[4] 645 = the number of children that were supposed to be given ≥ 2 doses of PCV13 when they were older than 10 weeks or two and a half months.

[5] Fever = 37.5-39.0°C

[6] Neonatal problems = neonatal hospitalisation, neonatal asphyxia or neonatal resuscitation

[7] Other diseases = genetic disorders (sickle cell disease n= 2, Down syndrome n=1), cerebral palsy n=3
